# Mesenchymal stromal cells derived from whole human umbilical cord exhibit similar properties to those derived from Wharton's jelly and bone marrow

**DOI:** 10.1002/2211-5463.12104

**Published:** 2016-10-08

**Authors:** Claire Mennan, Sharon Brown, Helen McCarthy, Eleni Mavrogonatou, Dimitris Kletsas, John Garcia, Birender Balain, James Richardson, Sally Roberts

**Affiliations:** ^1^ The Robert Jones & Agnes Hunt Orthopaedic Hospital NHS Foundation Trust & Institute of Science & Technology in MedicineKeele UniversityOswestryShropshireUK; ^2^ Laboratory of Cell Proliferation and AgeingInstitute of Biosciences and ApplicationsNational Centre for Scientific Research “Demokritos”AthensGreece

**Keywords:** allogeneic cell therapy, bone marrow‐derived mesenchymal stromal cells, immunomodulation, umbilical cord‐derived mesenchymal stromal cells

## Abstract

Mesenchymal stromal cells (MSC) can be isolated from several regions of human umbilical cords, including Wharton's jelly (WJ), artery, vein or cord lining. These MSC appear to be immune privileged and are promising candidates for cell therapy. However, isolating MSC from WJ, artery, vein or cord lining requires time‐consuming tissue dissection. MSC can be obtained easily via briefly digesting complete segments of the umbilical cord, likely containing heterogenous or mixed populations of MSC (MC‐MSC). MC‐MSC are generally less well characterized than WJ‐MSC, but nevertheless represent a potentially valuable population of MSC. This study aimed to further characterize MC‐MSC in comparison to WJ‐MSC and also the better‐characterized bone marrow‐derived MSC (BM‐MSC). MC‐MSC proliferated faster, with significantly faster doubling times reaching passage one 8.8 days sooner and surviving longer in culture than WJ‐MSC. All MSC retained the safety aspect of reducing telomere length with increasing passage number. MSC were also assessed for their ability to suppress T‐cell proliferation and for the production of key markers of pluripotency, embryonic stem cells, tolerogenicity (CD40, CD80, CD86 and HLA‐DR) and immunomodulation (indoleamine 2,3‐dioxygenase [IDO] and HLA‐G). The MC‐MSC population displayed all of the positive attributes of WJ‐MSC and BM‐MSC, but they were more efficient to obtain and underwent more population doublings than from WJ, suggesting that MC‐MSC are promising candidates for allogeneic cell therapy in regenerative medicine.

**Abbreviations**
BM‐MSCs: bone marrow MSC
DT: doubling time
ESC: embryonic stem cells
HLA: human leukocyte antigen
IDO: indoleamine 2,3‐dioxygenase
IFN‐γ: interferon gamma
ISCT: international society for cell therapy
MC‐MSC: whole cord MSC
SSEA: stage‐specific embryonic antigen
TRA: tumour repressor antigen
UC‐MSC: umbilical cord mesenchymal stem cells
WJ‐MSC: Wharton's jelly MSC

Mesenchymal stromal cells (MSC) from umbilical cords are of increasing interest for cell therapy for many areas of regenerative medicine, including the treatment of degenerative musculoskeletal disorders, as they are well‐supported by medical ethics and are reported to contain immune privileged cells, rendering them potentially suitable for allogeneic therapies 1. The use of autologous cells for cell therapies has perceived advantages related to low risk of immune rejection. Nevertheless, cells taken from older patients, to treat age‐related musculoskeletal disorders such as arthritis may not grow well in culture 2 and patients often have to undergo two operations, the first to obtain the cells and the second to implant them. Allogeneic cell banks, providing an ‘off the shelf’ cell product, would alleviate the need for two surgical procedures. Furthermore, patients with a degenerate condition may have inherited defective genes in a particular cell type. Thus, a characterized allogeneic cell may have the potential of providing an improved genotype and so a better quality tissue than a defective autologous cell. The use of MSC as an allogeneic treatment in the clinic may only be possible if they proliferate well enough in culture at a low passage number to create cell banks. The proliferative capacity of MSC at low passage may be critical for forming useful cell banks since there are recent reports that BM‐MSC at high passage number (passage 5–10) may have diminished efficiency and reduced therapeutic effect *in vivo* 3. The mode of action of MSC remains to be confirmed, but rather than them differentiating *in vivo* into repair tissues, there is an increasing body of evidence from *in vitro* and *in vivo* studies suggesting that MSC function through trophic effects on endogenous cells as well as by secretion of immunomodulatory molecules 4, 5, 6. Indeed, Velthoven *et al*. 7 have shown that they may not survive long enough to differentiate at all.

It is also well known that the ability of MSC to modulate inflammatory processes or become ‘immunomodulating’ cells is enhanced by stimulation with proinflammatory cytokines, TNF‐α, IL‐1β and interferon‐γ (IFN‐γ) 8, 9. Indeed, the International Society for Cell Therapy (ISCT) working proposal advises that characterization of stem cells for therapy should include activation or ‘licensing’, which involves stimulation with IFN‐γ, either alone or with the addition of TNF‐α 8. The production of potent immunomodulating molecules, such as indoleamine 2,3‐dioxygenase (IDO), only occurs following cell activation with one or more of these inflammatory cytokines.

Due to their derivation from postembryonic tissue, MSC derived from umbilical cord exhibit some of the properties of embryonic stromal cells (ESC), but they also share characteristics with BM‐MSC derived from adult tissue. Unlike BM‐MSC, ESC do not up‐regulate MHC II/HLA‐DR molecules after stimulation with IFN‐γ 10. ESC are also well characterized as being pluripotent cells, that lack stage‐specific embryonic antigen (SSEA)‐1, and produce SSEA‐4, alkaline phosphatase, tumour repressor antigen (TRA)‐1‐60, TRA‐1‐81, OCT3/4, nanog and REX‐1. Reports of pluripotency and immunomodulatory capacity of UC‐MSC and BM‐MSC are more variable, possibly due to different culture conditions. In previous work our group isolated and characterized MSC from four distinct anatomical regions of the human UC: the umbilical vein, arteries, cord lining and WJ, and from a population isolated from the whole cord/mixed cord after enzyme treatment 11. All populations of cells were found to fit the MSC profile, according to the ISCT criteria. We did not identify any distinct differences between them except that cells isolated from MC and WJ showed slightly better differentiation potential than cells isolated from other cord regions, indicating MC‐MSC and WJ‐MSC to be the most promising candidates for regenerative cell therapies. In the light of new research showing that the mechanism of action may not be primarily through differentiation the focus of this study was to further characterize and examine potential immunomodulatory properties of MSC from WJ or whole human umbilical cord preparations, for a direct comparison of which cell type may be more suitable for regenerative cell therapies.

In addition to assessing telomere length and the presence of embryonic and pluripotent markers, we also analysed the immune properties of these cells before and after stimulation with the proinflammatory cytokine IFN‐γ and compared their responses to those of the well‐characterized BM‐MSC.

## Materials and methods

### Isolation and culture of cells from human umbilical cords and bone marrow

All samples were obtained after patients had provided informed consent; favourable ethical approval was given by the National Research Ethics Service (10/H10130/62). UCs were collected and processed within 24 h of natural delivery, as previously described 11. All cells were grown in ‘complete’ media containing Dulbecco's Modified Eagle's Medium (DMEM; F12), 10% fetal calf serum (FCS; Life Technologies, Paisley, UK) and 1% penicillin/streptomycin (P/S; Life Technologies). MC‐MSC were obtained by processing 2–3 cm of whole UC, which were weighed and minced into small pieces (~ 2 mm^3^) before digesting with 1 mg·mL^−1^ collagenase I (≥ 125 digesting units mg^−1^; Sigma‐Aldrich, Dorset, UK) for 1 h at 37 °C. Tissue was removed from the digest and the supernatant was centrifuged at 80 ***g*** for 10 min; the pellet was resuspended in 5 mL of medium and plated into a 25‐cm^2^ tissue culture flask (Sarstedt, Leicester, UK). WJ was dissected from approximately 6 cm of whole cord, weighed, minced and placed into a 25‐cm^2^ tissue culture flask for explant culture. Tissue was removed after 21 days of culture.

In addition, human BM‐MSC were obtained for comparison, from bone chips, harvested from the iliac crest of patients undergoing spinal fusion in the treatment for back pain (Table 1). Bone chips were perfused with complete medium; this perfusate (diluted 1 : 1 with medium) was then carefully layered over Lymphoprep (Fresenius Kabi Norge, Norway). Mononuclear cells were isolated after being centrifuged at 900 ***g*** for 20 min, resuspended in complete medium and centrifuged again at 750 ***g*** for 10 min. The resulting pellet was plated out in complete medium at a seeding density of 20 × 10^6^ cells per flask. After 24 h, nonadherent cells were removed by changing the medium and adherent cells were cultured in a monolayer. Medium was changed every 2–3 days. All cells were maintained in a humidified atmosphere at 5% CO_2_ and 21% O_2_ at 37 °C.

**Table 1:** Patient data for BM‐MSC, MC‐MSC and WJ‐MSC, showing the age of bone marrow donors and age of the mothers of umbilical cord donors**.**

Patient	BM‐MSC Age	Used for		
1 BM	29	T FC MLR		
2 BM	31	IDO FC MLR		
3 BM	32	FC		
4 BM	42	FC MLR		
5 BM	48	IDO HLA‐G		
6 BM	50	HLA‐G		
7 BM	66	HLA‐G		
8 BM	70	HLA‐G		
9 BM	79	IDO		

Patient	MC‐MSC Age	Used for	WJ‐MSC Age	Used for
1	19	T	19	T HLA‐G FC
2	22	IDO HLA‐G MLR	22	IDO HLA‐G MLR
3	26	FC	26	IDO FC MLR
4	31	IDO	NT	
5	33	T HLA‐G FC MLR	33	T HLA‐G FC
6	39	FC	39	IDO FC MLR
7	39	IDO HLA‐G FC MLR	NT	

The patients’ cells used in each experiment are indicated as follows: T, Telomere length; FC, flow cytometry (including pluripotency markers, costimulatory markers and HLA‐DR); IDO, RT qPCR data for IDO expression; HLA‐G, flow cytometry for HLA‐G; MLR, T cell and MSC coculture experiment; NT, not tested.

### Calculation of doubling time

To calculate doubling time (DT), cells were harvested, counted and replated when they reached 70% confluency. Doubling time was calculated using the formula DT = (*t*2 − *t*1)ln (2)/ln (*n*2/ *n*1) where *n*2 is the cell number at harvesting, *n*1 is the cell number at plating, *t*2 is the time at cell harvest and *t*1 is the time at plating 12.

### Analysis of telomere length

DNA was extracted from MC‐MSC, WJ‐MSC and BM‐MSC every third passage using the High Pure PCR Template Preparation Kit (Roche, Sussex, UK) and stored at −20 °C until needed. DNA content was measured using a NanoDrop (Fisher Scientific, Loughborough, UK). The TeloTAGGG kit (Roche) was used to determine the length of telomeres from MC (*n* = 2), WJ (*n* = 2) and BM‐MSC (*n* = 1) over several passages according to the manufacturer's instructions. Genomic DNA (1 μg) from each sample population was digested with a *Hin*fI/*Rsa*I mixture for 2 h at 37 °C and then loaded onto a 0.8% agarose gel. The DNA fragments were separated by gel electrophoresis for 2–4 h at 70 V and transferred to a nylon membrane (Macherey‐Nagel, Düren, Germany) by Southern blotting.

The blotted DNA fragments were hybridized to a digoxigenin (DIG)‐labelled probe specific for telomeric repeats and incubated with a DIG‐specific antibody covalently coupled to alkaline phosphatase, which was visualized by the chemiluminescence substrate CDP‐*Star*. The telomere bands were then demonstrated by exposing the blot to an X‐ray film at room temperature for 15–20 min and the average terminal restriction fragment (TRF) length was determined by comparing the signals relative to the molecular weight standard.

### Immunocytochemistry

The presence of pluripotency markers was assessed on MC‐MSC (*n* = 4), WJ‐MSC (*n* = 4) and BM‐MSC (*n* = 4) using antibodies against human OCT3/4 (Becton Dickinson & Company, Oxford, UK), nanog (R&D Systems, Oxford, UK) and REX‐1 (Abcam, Cambridge, UK). Cells were seeded onto chamber slides at a density of 5000 cm^−2^_,_ allowed to adhere overnight and then fixed with 4% paraformaldehyde for 20 min. Slides were washed twice with PBS before the addition of blocking buffer made up of 1% BSA, 0.1% Triton X‐100 and 10% normal serum of the appropriate species (i.e. donkey for nanog, goat serum for OCT3/4 and rabbit for REX‐1) in PBS for 1 h at room temperature. Slides were washed twice in PBS before adding the primary antibodies against OCT3/4 (1 : 1000; (mouse IgG1 monoclonal), nanog (1 : 50; goat IgG polyclonal) and REX‐1 (1 : 1000; rabbit IgG polyclonal) in the appropriate blocking buffer (containing the relevant serum above) and incubating overnight at 4 °C. The primary antibodies were removed and the slides were washed twice with PBS.

The relevant fluorophore‐labelled secondary antibody (donkey anti‐(goat IgG) Alexa Fluor 488, goat anti‐(mouse IgG) Alexa Fluor 488 or goat anti‐rabbit Alexa Fluor 488) was diluted (1 : 250) in blocking buffer and added to the cells, which were then incubated in the dark for 1 h at room temperature. Negative controls were obtained by using appropriate isotype antibodies or PBS in place of primary antibodies. Slides were washed twice with PBS before 4′, 6‐diamidino‐2‐phenylindole (Vector Laboratories, Peterborough, UK) stain was added to the cells as a counterstain to visualize cell nuclei, and the slides were then mounted and viewed under a Leica DMLB fluorescent microscope (Milton Keynes, UK). The H9 ESC cell line was used as a positive control for the production of OCT3/4, nanog and REX‐1, as previously described 13.

### Stimulation of cells with IFN‐γ

Human IFN‐γ (Promokine, Heidelberg, Germany) was used to stimulate cells at a concentration of 25 ng·mL^−1^ 14, 15. It was added to the growth media of MC‐MSC (*n* = 4), WJ‐MSC (*n* = 4) and BM‐MSC (*n* = 4) cultured in monolayer at 37 °C for 48 h, after which time cells were assessed for the production of costimulatory markers (CD40, 80 and 86), HLA‐DR and HLA‐G, by flow cytometry and IDO by western blot and RT‐qPCR.

### Immunoprofiling

Flow cytometry was used to assess the immunoprofile of UC‐MSC (*n* = 4) and BM‐MSC (*n* = 4). Cells at passage 3 were harvested, filtered through a 70‐μm mesh cell strainer, pelleted, resuspended in 2% bovine serum albumin (BSA) in PBS and counted. Around 100 000 cells were used for each antibody and the control. Cells were stained with antibodies against SSEA‐1, SSEA‐4, alkaline phosphatase, TRA‐1‐60 and TRA‐1‐81 and then labelled with a secondary antibody conjugated to FITC (SouthernBiotech, Cambridge, UK). Costimulatory markers were all detected with phycoerythrin (PE)‐conjugated antibodies against CD40, CD80 and CD86 (Becton Dickinson & Company). Antibodies to MHC II/HLA‐DR were also PE conjugated (ImmunoTools, Friesoythe, Germany). Appropriately isotype‐matched antibodies were used as negative controls in all analyses and embryonic stem cells (ESC) were used as a positive control for these. The presence of HLA‐G was also assessed using an anti HLA‐G antibody (Santa‐Cruz, Dallas, TX, USA), both on the cell surface and internally, using a Cytofix/Cytoperm^™^ plus Fixation/Permeabilisation Kit (Becton Dickinson & Company) according to the manufacturer's instructions. Cells were analysed on a FACSCanto II flow cytometer using diva 7 software (Becton Dickinson & Company). The human choriocarcinoma JEG3 cell line (ECACC, Salisbury, UK) was used as a positive control for HLA‐G.

### Western blotting analysis of IDO

Cells were lysed by the addition of cold lysis buffer (0.005% Tween 20, 0.5% Triton X‐100 at 4 °C) containing a general protease inhibitor cocktail (Sigma‐Aldrich) at 1 mL per 10 × 10^6^ cells. Cell lysates were frozen and stored at −20 °C until needed. Total protein concentrations were determined using the bicinchoninic acid assay (Life Technologies) following the manufacturer's protocol, to ensure equal protein loading onto the gel for each sample.

Western blotting was used for the detection of IDO in whole cell lysates of MC‐MSC, WJ‐MSC and BM‐MSC. Electrophoresis was performed under reducing conditions by loading a 20‐μg protein sample into each well of a ready‐made precast NuPAGE 15‐well Bis‐Tris Mini 4–12% gradient gel (Life Technologies). Proteins were then electroblotted onto nitrocellulose membranes using the iBlot system and iBlot gel nitrocellulose transfer stacks (Life Technologies). Antibody detection was carried out using iBlot western detection stacks and iBlot western detection chromogenic kits (Life Technologies). Briefly, the primary (monoclonal) antibodies against IDO (Abcam, Cambridge, UK; 1 : 500) were applied using the iBlot apparatus, followed by a secondary antibody (1 : 500) (Life Technologies) conjugated to horseradish peroxidase. Blots were then washed with Invitrogen wash solution three times, followed by a further two washes in autoclaved water. Finally the chromogenic substrate Novex^R^ Alkaline Phosphatase (Life Technologies) was applied to the membrane and colour development carried out for a maximum of 1 h at room temperature.

### Reverse transcriptase‐quantitative PCR

RNA was extracted from MC‐MSC, WJ‐MSC and BM‐MSC before and after exposure to IFN‐γ using the RNeasy Mini kit (Qiagen, Sussex, UK), following the manufacturer's instructions. RNA was eluted from the spin column with RNAse free water and stored at −80 °C until RT‐qPCR analysis for IDO was performed using the SYBR green mastermix (Applied Biosystems, Warrington, UK) with GAPDH as a reference gene (Qiagen, QuantiTect Primer Assay). The reaction was conducted in the ABI 7500 RT‐qPCR system (Applied Biosystems) at 95 °C for 10 min followed by 40 cycles of 95 °C for 15 s then 60 °C for 1 min and data were captured using the sds software (Applied Biosystems). The presence of the IDO mRNA in IFN‐γ‐stimulated cells was expressed as a ratio compared to unstimulated cells, using the comparative threshold method 16. A twofold change threshold (up‐ or down‐regulated) was deemed biologically significant. IDO gene expression was measured over a time course of exposure to IFN‐γ between 1 and 48 h, with the same time point representing the control in normal medium without the addition of the inflammatory cytokine. IDO mRNA levels were initially normalized to the reference gene before calculating the ratio of mRNA in the stimulated versus unstimulated cells.

### T cell isolation and co‐culture with MSC

Human naïve CD4^+^ T cells were isolated from heparinized blood using the naïve CD4^+^ isolation kit II (Miltenyi Biotech, Cologne, Germany). For this, peripheral blood mononuclear cells (PBMCs) were isolated by density gradient centrifugation at 900 ***g*** for 20 min over Lymphoprep. The buffy coat layer was resuspended in cold PBS and washed several times to remove platelets. Cells were counted and re‐suspended in buffer composed of 2 mm EDTA (in 0.5% BSA) in PBS. Naïve CD4^+^ T cell Biotin‐Antibody Cocktail II was added and the cells were incubated for 10 min at 4 °C. The cells were then resuspended in 1–2 mL of EDTA buffer and centrifuged. Supernatant was aspirated and the cells were resuspended in EDTA/BSA buffer before adding antibiotin microbeads, which were incubated for 15 min at 4 °C. The cells were then washed and resuspended in EDTA/BSA buffer prior to cell sorting via negative selection using a MACS column and separator magnet (Miltenyi Biotech) as detailed in the manufacturer's instructions. T cells were then labelled with Violet Proliferation Dye 450 (VPD450; BD Horizon^™^, Oxford, UK) according to the manufacturer's instructions.

MC‐MSC (*n* = 3), WJ‐MSC (*n* = 3) and BM‐MSC (*n* = 3) were seeded into separate 24‐well plates at a density of 10 000 cells per well. CD4^+^ T cells were added at a density of 100 000 cells per well to give a ratio of MSC:T cells of 1 : 10. Allogeneic stimulator human PBMCs from healthy donors were added at the same density with T cells and all cells were cultured at 37 °C in a humidified atmosphere for 5 days; after this time T cells were removed via gentle pipetting and analysed via flow cytometry using the violet laser for analysis of the VPD450 dye and calculation of T‐cell proliferation. The T‐cell response was analysed using the proliferation platform in FlowJo (Tree Star, Ashland, OR, USA). The percentage of dividing cells as well as the average number of cell divisions was calculated using the proliferation platform. Controls consisted of T cells alone and T cells with stimulator PBMCs, to check for a lack of division and T‐cell division without suppression respectively. All experiments were conducted in triplicate.

### Statistics

**Figure 1:**
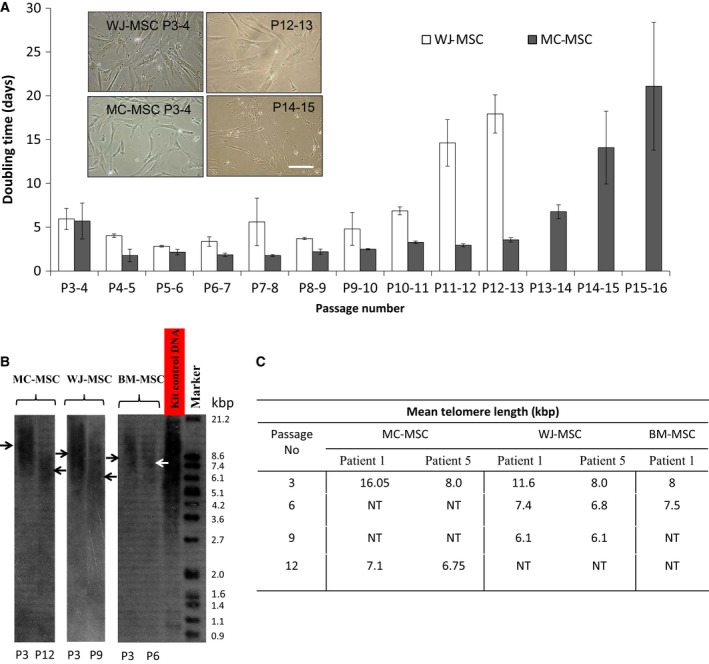
Doubling time, cell morphology and telomere length of MC‐MSC and WJ‐MSC over extended culture. (A) Doubling time and representative cell morphology of MC‐MSC and WJ‐MSC over an extended cell culture period *in vitro*. (*n* = 3 for MC‐MSC and WJ‐MSC; scale bar represents 100 μm). Bars represent average doubling times at sequential passages ± SEM. (B) Telomere length of MC‐MSC, WJ‐MSC (data shown from patient 1) and BM‐MSC (patient 1) in culture at varying passage numbers. Black and white arrows indicate the positions of the terminal restriction fragments of the telomeres on the gel. (C) Mean telomere length (kbp) for MC‐MSC, WJ‐MSC and BM‐MSC at different passage numbers, showing shortening of telomeres with increasing time in culture. NT, not tested. Donor ages for cells used in the telomere experiment were as follows: BM‐MSC Patient 1, 29 years, MC‐MSC and WJ‐MSC Patient 1, 19 years, Patient 5, 33 years.

**Figure 2:**
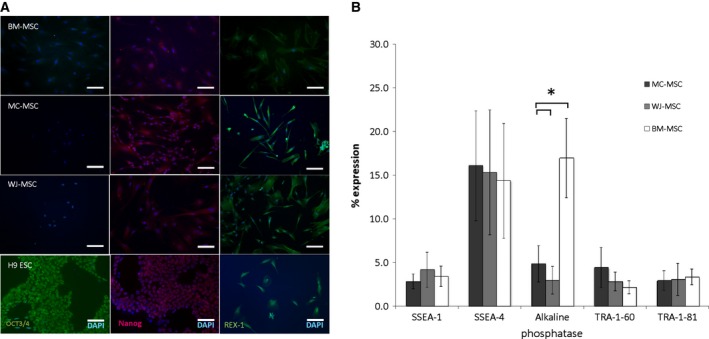
Immunocytochemistry and flow cytometry showing the presence of pluripotency markers and stage‐specific embryonic antigens on BM‐MSC, MC‐MSC and WJ‐MSC. (A) Immunocytochemical staining of BM‐MSC, MC‐MSC, WJ‐MSC and H9 ESC cell line with the pluripotency markers, OCT3/4, nanog and REX‐1. Scale bars represent 100 μm. (B) The presence of stage‐specific embryonic antigens (SSEA)‐1 and 4, tumour repressor antigens (TRA)‐1‐60, TRA‐1‐81 and the pluripotency marker alkaline phosphatase on MC‐MSC, WJ‐MSC and BM‐MSC, assessed by flow cytometry. *n* = 4 for each cell type. Bars represent average percentage of positively stained cells ± SEM. The ESC H9 cell line was also used as a positive control for the markers shown in B (data not shown). Levels of significance indicated are *P < 0.05.

**Figure 3:**
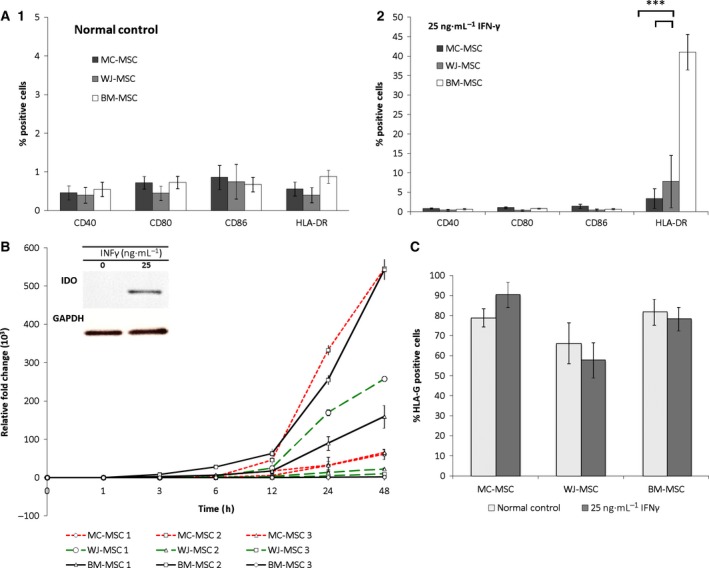
Assessment of the production of costimulatory markers, HLA‐DR and immunomodulatory proteins on BM‐MSCs, MC‐MSCs and WJ‐MSCs before and after stimulation with IFN‐γ. (A.1) Assessment of the production of the costimulatory markers (CD40, 80, 86) and MHC class II/HLA‐DR on MC‐MSC, WJ‐MSC and BM‐MSC (*n* = 4 for each cell type) cultured in normal media without IFN‐γ and (A.2) following stimulation with 25 ng·mL^−1^ IFN‐γ for 48 h as assessed by flow cytometry. (B) Representative western blot showing the production of IDO and the reference gene GAPDH in MC‐MSC before and after stimulation with IFN‐γ for 48 h. The graph shows a 48‐h time course for the up‐regulation of IDO after stimulation with IFN‐γ in MC‐MSC, WJ‐MSC and BM‐MSC, analysed by RT qPCR. (C) Production of HLA‐G, assessed by flow cytometry, on MC‐MSC, WJ‐MSC and BM‐MSC cultured in normal media with no inflammatory stimulus and media with 25 ng·mL^−1^ IFN‐γ (*n* = 4). Graphs present the average of values ± SEM. Levels of significance indicated are ***P < 0.001.

graphpad prism (GraphPad Prism, San Diego, CA, USA) was used for statistical analysis. Data are presented as mean ± standard error mean (SEM) and mean ± standard deviation (SD) in the graphs and text respectively. The two‐way ANOVA and multiple *t*‐test was used for growth kinetics analysis (Fig. 1A). Flow cytometry data showing the presence of pluripotency markers on MSC (Fig. 2B) and markers with and without IFN‐γ stimulation (Fig. 3) were analysed using two‐way ANOVA with Bonferroni's multiple comparisons test. Levels of significance are indicated as *P < 0.05, **P < 0.01 and ***P < 0.001.

## Results and Discussion

### Longevity and telomere length

The mean doubling time for MC‐MSC (*n* = 3) was 2–3 days up to passage 12–13, where growth slowed markedly, reducing to 20 days at passage 15–16 when experiments were stopped. In contrast, WJ‐MSC (*n* = 3) showed significantly slower growth (*P* = 0.001 at P10‐11, *P* = 0.01 at P11‐12 and *P* = 0.02 at P12‐13), with mean doubling times of 4–5 days up to passage 10–11 and 18 days at passage 11–12. Both MC‐MSC and WJ‐MSC appeared larger and more spread at higher passages at the point cell growth started to slow (Fig. 1A), which may be indicative of senescence 17. Previous work in our group showed BM‐MSC had longer doubling times than these UC populations of MSC, of 5–6 days at passage 2 and 12 days at passage 3 18. While longer doubling times of BM‐MSC compared to UC‐MSC has also been reported by other groups 14, our doubling times may be longer than that reported by other centres, since our group has not added any specific growth factors. Many groups add growth factors such as fibroblastic growth factor‐2 (FGF‐2) to supplement BM‐MSC culture medium, which is known to enhance their proliferation. FGF‐2 is secreted by MSC in culture but becomes depleted during serial passage *in vitro* if not supplemented 19, 20. Another possible cause of observed differences between BM‐MSC and other MSC tested in this work could be due to the fact that BM‐MSC were obtained from older patients undergoing spinal fusion for back pain. In future work, BM from younger female donors may be a better source of MSC for comparison.

Limited proliferative capacity could have implications in cell therapies that require high cell doses per kg of patient weight. For example, up to 10 million cells per kg of patient weight may be infused at several time‐points for the treatment of graft‐versus‐host‐disease (GVHD) with allogeneic MSC 21. The differences in growth rate between MC‐MSC and WJ‐MSC shown in Fig. 1A may reflect heterogeneity of cells in cultures of MC‐MSC, with a combination of different (sub) populations of cells possibly supporting better growth, in contrast to cells isolated from a single tissue source such as WJ. The short (1 h) enzymatic digestion of sections of whole cord provided 6 × 10^5^ (± 2.4 × 10^4^) cells per gram of cord (*n* = 6) at passage 0–1. This is in accordance with Marmotti *et al*. 22 who retrieved 6.6 × 10^5^ cells per gram UC, although that study used minced umbilical cord fragments in explant culture rather than enzymatic digest. Furthermore, the average time taken from passage 0 to 1 was 8.8 days shorter for MC‐MSC compared to WJ‐MSC, with MC‐MSC reaching passage 1 in an average time of 19.4 days ± 0.41 (*n* = 9), whereas WJ‐MSC took 28.2 ± 0.48 days (*n* = 9) and yielded approximately 1.1 × 10^6^ (± 2.5 × 10^4^) cells per gram WJ (*n* = 5). BM‐MSC were seeded at an average cell density of 20.2 × 10^6^ (± 4.5 × 10^5^) (*n* = 5) cells per 75 cm^2^ and took 20.6 ± 2.14 days to reach passage 1. Approximately 1.4 × 10^6^ (± 2.0 × 10^5^) BM‐MSC were obtained from each sample of bone chip wash out treated with Lymphoprep.

If cord cells are to be used to regenerate tissues then it is important that sufficient cells are grown from a single cord to allow full characterization, but with a population growth that is also limited and nontumorigenic and so likely to be safe. If all the MC‐MSC were used from a single cord which weighs an average of 40 g, taking into account the above yield of 0.6 million cells per gram of cord, we estimate that 8 × 10^14^ cells could be generated by passage 10. As an example of the number of cells used in a clinical therapy, we currently use approximately 4 × 10^6^ cells per patient to treat chondral defects with a mean size of 4 cm^2^. Thus, 2 × 10^8^ doses could be used from one cord, which would therefore be 22 times more than required to treat the (estimated) 8.75 million people in the UK who have OA.

Studies trying to determine whether *in vitro* expansion impacts the genomic stability of MSC have shown that umbilical cord MSC senesce in culture at high passage 23, which we suggest is a desirable trait.

We found that MC‐MSC (*n* = 3), WJ‐MSC (*n* = 3) and BM‐MSC (*n* = 1) all undergo telomere shortening during culture and with increasing passage number (Fig. 1B, C). BM‐MSC had a mean telomere length of 8 kpb at passage 3, which is in accordance with Marmotti *et al*. 24 who reported that BM‐MSC from six patients aged 20–30 years had telomere lengths in the range of 7.8–9.8 kbp. This is the same as that measured from matched samples of MC‐MSC and WJ‐MSC from the same cord (patient 5, 33‐year‐old mother) at passage 3. However, the MC‐MSC from the cord of baby from a 19‐year‐old mother (patient 1) had longer telomeres at passage 3 (16.1 kpb) indicating individual variation in telomere length. It is unknown if maternal age could influence telomere length in fetal tissues. Paternal age has been shown to be significantly and positively associated with telomere length of both male and female offspring 25. Nordfjäll *et al*. 26 showed that telomere length is documented to have a hereditary component, with both paternal and x‐linked inheritance being proposed. However, large variations in telomere length between individuals of the same age have been shown in other studies 27 and could account for the differences seen between cells from different individuals in this study. Similarly, Wang *et al*. 23 reported that although UC‐MSC demonstrated shortening of their telomeres with increasing passage number, they also showed a degree of genomic instability over extended time in culture but did not undergo malignant transformation in small animal models. This is important from a safety point of view as the ability to maintain telomere length has been seen in cancer cells and germ cells and has been attributed to the cells’ ability to produce telomerase 28. Vidal *et al*. 29 reported that UC‐MSCs have long telomere sequences and a greater expansion capability than BM‐MSC, suggesting a late onset of senescence of this cell population during *in vitro* expansion.

### Pluripotency and expression of stage‐specific markers

Results of immunocytochemistry for the embryonic stromal cell markers, OCT3/4, nanog and REX‐1, which are critical for both self‐renewal and maintenance of an undifferentiated state are shown for the MSC populations in Fig. 2A. None of the MSC, whether sourced from MC (*n* = 4), WJ (*n* = 4) or BM (*n* = 4) showed positive staining for OCT3/4. However, differences were seen in the production of nanog and REX‐1 between cell populations. BM‐MSC showed the least staining for nanog and REX‐1 with MC‐MSC showing the strongest staining for both. As REX‐1 is also a marker of proliferative capacity and MC‐MSC showed shorter doubling times, it was expected that the intensity of REX‐1 staining would be higher in these cells. OCT3/4, nanog and REX‐1 are transcriptional activators, which can act together in concert to retain self‐renewal and prevent differentiation. Reports differ on the presence of OCT3/4 on UC‐MSC and BM‐MSC, which is thought to be attributable to different culture conditions 30. OCT3/4 can both activate and repress REX‐1, which implies a dual regulatory ability. Nanog has also been shown to be a transcriptional activator of REX‐1 and helps sustain its expression. MSC from both UC and adipose tissue have been found to express high levels of REX‐1 and to proliferate rapidly in culture 31. Our results support the findings that BM‐MSC produce low levels of REX‐1; others have also shown similar results and linked REX‐1 production to the proliferative state of the cell 31. The presence of other markers indicative of pluripotency, which are commonly expressed by ESC was assessed by flow cytometry. All MSC populations expressed low levels of SSEA‐1, TRA‐1‐60 and TRA‐1‐81 (Fig. 2B). Approximately 12–15% of all populations were positive for SSEA‐4 with no significant difference between MSCs from different localities (*P* = 0.97), whereas significantly more BM‐MSC were positive for alkaline phosphatase than MC‐MSC or WJ‐MSC (*P* = 0.02, Fig. 2B). SSEA‐4 has been shown previously to be expressed by adult BM‐MSC 14, 32, 33, although the reasons for adult MSC expressing embryonic markers remain unclear.

The alkaline phosphatase antibody used in this study reacts with the tissue nonspecific (TNS) isoform of alkaline phosphatase which is expressed at high levels in undifferentiated pluripotent stem cells 34 such as induced pluripotent stem cells and ESC. Levels of TNS alkaline phosphatase decrease on differentiation (except if MSCs differentiate to bone), therefore this antibody is often used to monitor the differentiation status of pluripotent stem cells. However, it is of note that as the isozyme of alkaline phosphatase is also the same as that expressed by bone cells 35, it is also considered to be a general osteoblast marker 36, 37. Therefore, the higher production seen in BM‐MSC in this study may be explained by their higher propensity to differentiate to bone. This is further supported by other studies showing that early osteogenic differentiation of BM‐MSC relates to a significant increase in TNS alkaline phosphatase production 38. Kim *et al*. 38 also found that populations of BM‐MSC that were negative for alkaline phosphatase, expressed higher levels of REX‐1 and nanog, as estimated using RT‐qPCR, and were able to differentiate into multiple cell types better than their alkaline phosphatase‐positive counterparts. The implication is that alkaline phosphatase‐negative MSC retain a more primitive phenotype, which may indicate that UC‐MSC with their lower production of alkaline phosphatase are more primitive than BM‐MSC.

#### Production of immunomodulatory proteins before and after cell ‘licensing’

All cells were negative for the costimulatory markers, CD40, CD80, CD86 and HLA‐DR in normal medium without IFN‐γ (Fig. 3A1). Significantly more BM‐MSC (*n* = 4) were positive for HLA‐DR (41 ± 9%, *P* < 0.001) after IFN‐γ stimulation than MC‐MSC (*n* = 4) (3 ± 5%) or WJ‐MSC (*n* = 4), the proportion of which was the same ± IFN‐γ (8 ± 13%; Fig. 3A2). None of the MSC populations produced IDO without IFN‐γ stimulation, as shown by western blot and RT‐qPCR analyses (Fig. 3B). However, following the addition of 25 ng·mL^−1^ IFN‐γ, IDO was up‐regulated by all cell populations within an hour of IFN‐γ stimulation and continued to rise to the 48‐h time point, although there were large differences between individuals.

Costimulatory markers, such as CD40, 80 and 86, and HLA‐DR are expressed on professional antigen‐presenting cells (APCs) of the immune system but are obviously undesirable on cells destined for cell therapies. CD40 is produced on dendritic cells, macrophages and B cells, but it can also be found on other cell types such as endothelial cells, tumour cells and fibroblasts 39, 40. In the presence of IFN‐γ, BM‐MSC up‐regulate HLA‐DR and have been found to be capable of functioning as APCs. In addition, they have also exhibited phagocytic properties reminiscent of professional immune cells 41. Although an inflammatory stimulus, for example, IFN‐γ, up‐regulates HLA‐DR in BM‐MSC, costimulatory molecules are not produced 6, 14, 42. If both HLA‐DR and costimulatory molecules were to be present, an undesirable immune response could result 43, which may make these cells unsuitable for allogeneic use.

Indoleamine 2,3‐dioxygenase was up‐regulated at the 48‐h time point post IFN‐γ stimulation for all MSC populations studied but to varying degrees within the cell source and individual. BMSC showed 1.4 × 10^3^, 460 × 10^3^ and 159 × 10^3^ fold up‐regulation, MC‐MSC 65 × 10^3^, 61 × 10^3^ and 393 × 10^3^ fold, and WJ‐MSC 9.5 × 10^3^, 22 × 10^3^ and 273 × 10^3^ fold (three patients per cell type). Other studies have also reported variable up‐regulation of IDO between donors and suggest that the reason for this is the existence of an intrinsic variation in responsiveness and plasticity of MSC to inflammatory cytokines 6. This suggests that it would be important for patients’ cells destined for allogeneic cell banks to have their immunomodulatory potential tested prior to banking, to assess the response to inflammatory cytokines for each donor. Table 1 shows the ages of all MSC donors used in this work, differences in IDO expression could not be correlated with patient age as donor ages for BM‐MSC (up‐regulating the highest to the lowest IDO) were 79, 31 and 48 years old respectively. For MC‐MSC, maternal ages were 39, 31 and 22 years old, and for WJ‐MSC, again from highest to lowest IDO expression, 22, 36 and 39 years old.

HLA‐G (Fig. 3C) was constitutively expressed by all cell types regardless of inflammatory stimulus, with the mean percentage of cells expressing positivity being 79 ± 9% for MC‐MSC, 66 ± 20% for WJ‐MSC and 82 ± 12% for BM‐MSC, without IFN‐γ stimulation. There was no significant difference following IFN‐γ stimulation, with 90 ± 12% of MC‐MSC, 58 ± 17% of WJ‐MSC and 78 ± 11% of BM‐MSC being positive in the presence of IFN‐γ. HLA‐G is an important immunomodulatory molecule, which has receptors on many subsets of immune cells and it is capable of inducing apoptosis of activated natural killer (NK) cells, CD4^+^ and CD8^+^ T cells. HLA‐G is classically associated with protection of the foetus from maternal uterine NK cells. It is a ligand for NK cell inhibitory receptor (KIR2DL4) and therefore its production on the trophoblast cells of the placenta defend against NK cell‐mediated death 44. The production of HLA‐G on MSC is likely to confer therapeutic benefit, by evading detection and destruction by the hosts’ immune system. Cells producing high levels of HLA‐G may also have the potential to calm inflammation 45, such as that which may be found in a degenerate or osteoarthritic joint 46. Although IDO and HLA‐G have been tested as immunosuppressive factors in this study, there are many others that are produced by MSC which may offer a potential therapeutic benefit. For example, transforming growth factor‐ β (TGF‐β), IL‐10, Prostaglandin E_2_ (PGE_2_) and tumour necrosis factor‐inducible gene 6 protein (TSG6) have all been shown to have a role in reducing inflammation 42, 47, 48.

### T‐cell proliferation assays

**Figure 4:**
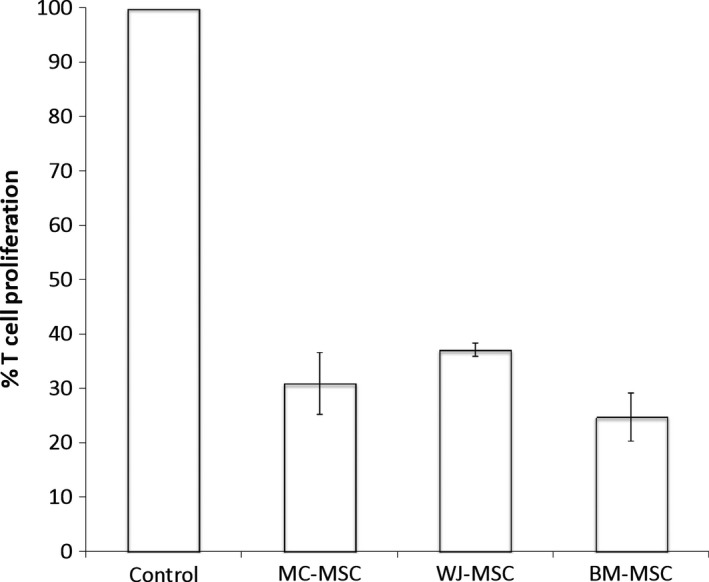
The suppression of T‐cell proliferation by resting MC‐MSC, WJ‐MSC and BM‐MSC. Naïve CD4^+^ T cells were labelled with violet proliferation dye and stimulated with allogeneic stimulator PBMCs (at a ratio of 1 : 1) in coculture with either MC‐MSC, WJ‐MSC and BM‐MSC (T cell to MSC ratio of 10 : 1) for 5 days prior to flow cytometry analysis of the T cells to assess the proliferative response. Each reaction was done in triplicate. Data are expressed relative to T cells and allogeneic stimulator PBMCs alone, without addition of MSC (assigned to 100% proliferation). The control shows T‐cell proliferation stimulated with allogeneic PBMCs alone without the addition of MSC. Data are shown as average percentage of T‐cell proliferation ± SEM. *n* = 3 for each cell type.

Due to the large amount of literature available on the suppression of activated T cells and mixed lymphocytes by BM‐MSC and UC‐MSC, a simple coculture method was used to determine if there were any differences between the populations of MSC examined in this study (MC‐MSC, WJ‐MSC and BM‐MSC) on T‐cell proliferation. Since cells currently used for regenerative medicine, for example, autologous chondrocyte implantation are applied after being cultured ‘unprimed’; we tested unprimed MSC without exposure to IFN‐γ for the ability to suppress T‐cell proliferation. Figure 4 shows that all MSC suppress proliferation of T cells with no significant difference between the cell populations. MC‐MSC (*n* = 3) suppressed T‐cell proliferation by 69 ± 5%, WJ‐MSC (*n* = 3) by 63 ± 1% and BM‐MSC (*n* = 3) by 75 ± 4%. It is well known that MSC do not elicit a T‐cell response *in vitro,* and possess the capability to suppress activated T cells (CD4^+^ and CD8^+^), NK cells and B cells *in vitro* 14, 49, 50. The mechanisms of action are thought to be through paracrine effects (via immunomodulatory proteins such as HLA‐G and IDO) and through cell‐to‐cell contact 47.

Stimulated CD4^+^ T cells produce IFN‐γ as part of the immune response (or in this case after stimulation with allogeneic PBMCs) 51, 52, 53. Hence, it is likely that the effects of the coculture of BM‐MSC or UC‐MSC on the suppression of T cells are due to the effects of HLA‐G (which is produced by resting and primed MSC) 54 and immunomodulatory proteins produced by MSC after T cell produced IFN‐γ. Other studies show further enhancement of MSC‐mediated T‐cell suppression by priming MSC with IFN‐γ prior to coculture with activated T cells or mixed lymphocytes 14, 55.

## Conclusion

The retrieval of sufficient numbers of MSC from aged patients for autologous cell therapy can be challenging and may be further complicated by donor site morbidity and painful harvesting procedures. In addition, autologous therapy may be inappropriate for some patients if they have a particular genetic make‐up, which is defective in terms of regeneration or maintenance of the appropriate extracellular matrix. UC‐MSC may offer an alternative to autologous therapies as they are easily sourced and expanded from waste tissue. However, reports in the literature vary on the characterization of cells isolated from the distinct anatomical regions of the human UC and often favour the use of MSC isolated from WJ. In this study, we have applied an easy and rapid method to collect an adequate number of MC‐MSC (0.6 million cells per gram cord tissue at passage 1), by simple mincing of the UC followed by a short (1 h) enzymatic digestion. Conversely, to source WJ‐MSC requires a more lengthy tissue dissection and 8.8 days longer in culture than MC‐MSC to reach passage 1. Although much work has been done on the immunomodulatory capacity of MSC from bone marrow and WJ, there are few reports on cells isolated from whole umbilical cord. Our results show that MC‐MSC share many attributes with WJ‐MSC and BM‐MSC. However, they survive significantly longer in culture, proliferate faster and are easier to obtain, requiring minimal tissue manipulation and handling. This head‐to‐head comparison shows that MC‐MSC offer a valuable and readily available source of cells with potential use in regenerative medicine.

## Author contributions

CM carried out the isolation and growth of UC‐MSC and BM‐MSC, growth kinetics, immunocytochemistry, telomere length analysis, flow cytometry, RT‐qPCR, T cell coculture experiments and prepared the manuscript. SO helped with western blotting and preparation of the manuscript. HM helped with RT‐qPCR, analysis and manuscript preparation. EM and DK helped prepare the telomere length assay and helped in the manuscript preparation. JG participated in BM‐MSC stimulation experiments, helped with flow cytometry and performed statistical analysis. BB, JBR and SR participated in the design and co‐ordination of the study and helped to draft the manuscript. All authors read and approved the final manuscript. SR obtained funding for the study.
